# Possible gabapentin and ketamine interaction causing prolonged central nervous system depression during post-operative recovery following cervical laminoplasty: a case report

**DOI:** 10.1186/1752-1947-5-167

**Published:** 2011-04-28

**Authors:** Ali R Elyassi, Robert P Long, Robert P Bejnarowicz, Bruce A Schoneboom

**Affiliations:** 1 Tripler Army Medical Center, Department of Surgery, 1 Jarrett White Road, Honolulu, HI 96859-5000, USA

## Abstract

**Introduction:**

The drugs gabapentin and ketamine are used frequently in the peri-operative setting. There is poor documentation whether or not gabapentin and ketamine interact to cause prolonged depression of the central nervous system.

**Case Presentation:**

The following is a case report in which a patient, a 58-year-old African-American man, with a history of post-traumatic stress disorder and chronic pain underwent a cervical laminoplasty procedure. The patient presented post-operatively in a dissociative state with paralysis, anarthria and preservation of consciousness. All organic causes were excluded, with the exception of prolonged central nervous system depression from a gabapentin/ketamine drug interaction. A new onset conversion disorder could also not be excluded.

**Conclusion:**

Although this case by itself is not enough evidence to substantiate a true adverse reaction between gabapentin and ketamine, it is enough to warrant further investigation.

## Introduction

The incidence of post-traumatic stress disorder (PTSD) is undoubtedly on the rise, given the recent events of the World Trade Center and Pentagon terrorist attacks coupled with the current wars in Iraq and Afghanistan. The prevalence of PTSD in the United States population is estimated to be approximately 8%. According to the Veterans Health Administration (VHA), Operation Iraqi Freedom (OIF) and Operation Enduring Freedom (OEF) veterans seeking VHA health care have been diagnosed predominantly with PTSD [1,2].

PTSD can give rise to pain as suggested by the high rates of chronic pain in patients with a history of childhood abuse [3]. Gabapentin and ketamine have independently shown improvement in chronic pain associated with PTSD [3-5]. Unfortunately, however, there is little documented evidence to suggest that gabapentin and ketamine interact to additively or synergistically depress the central nervous system (CNS) and/or have respiratory-depressant effects, especially in elderly or debilitated patients [6].

The following is a case report in which a patient with a history of PTSD and chronic pain underwent a cervical laminoplasty procedure. Our patient presented post-operatively in a dissociative state with paralysis, anarthria (loss of articulate speech), and preservation of consciousness. All organic causes were excluded, with the exception of prolonged CNS depression from a gabapentin/ketamine drug interaction. A new onset conversion disorder could also not be excluded.

## Case presentation

**Figure 1:**
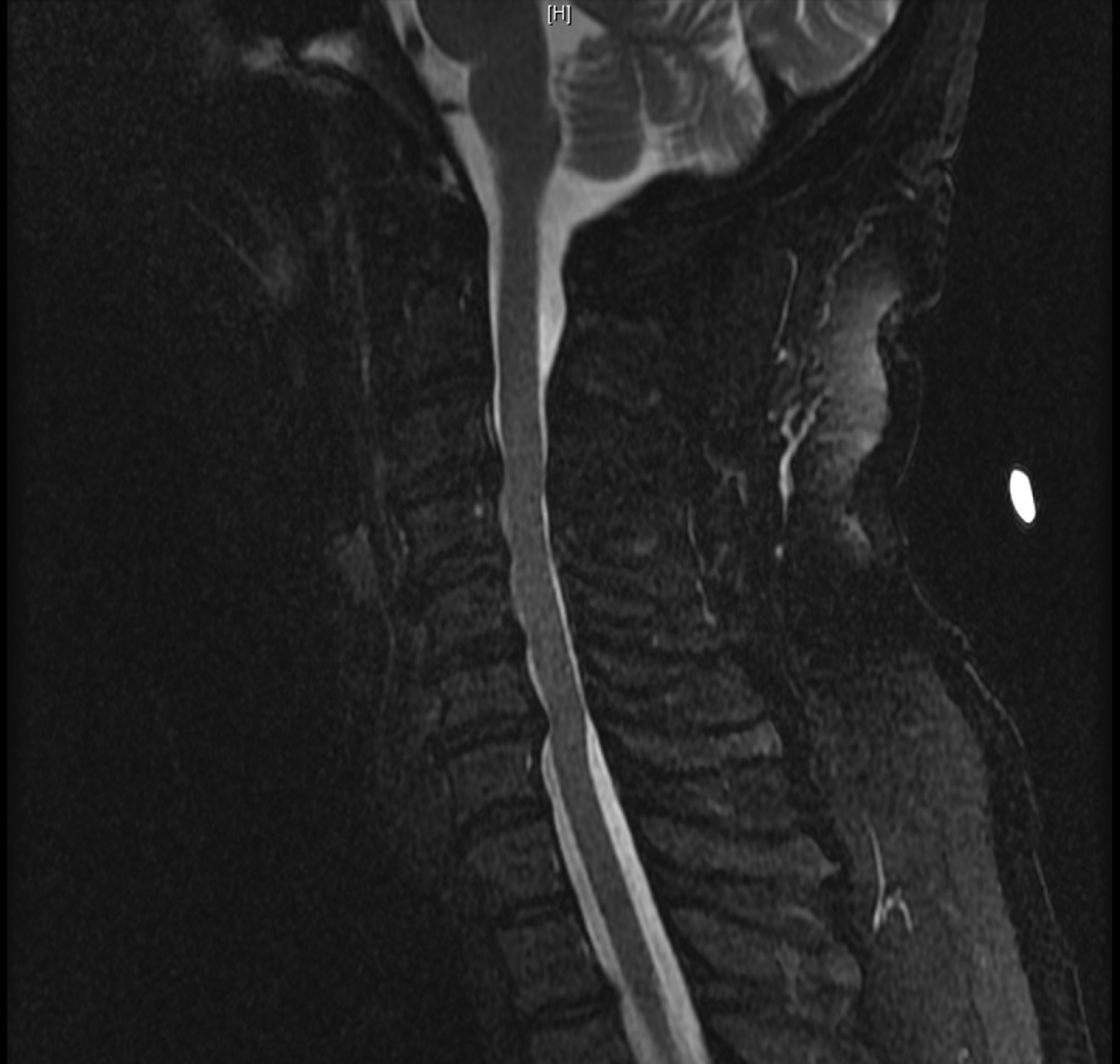
**Sagittal T2-weighted MRI showing narrowing of spinal canal at C3-C7**.

A 58-year-old African-American man presented to our neurosurgery clinic for cervical spinal stenosis. He had an approximate one-year history of slowly declining ability to ambulate, chronic pain in both hands, as well as mild paresthesias and decreased sensation involving fingers on both hands. Additionally, he described grip weakness, greater in his left than right. Both computed tomography (CT) and magnetic resonance imaging (MRI) revealed multi-level degenerative disc disease, causing narrowing of his spinal canal at cervical levels 3-7 (C3-C7) (Figure 1). Given our patient's clinical presentation and radiographic evidence of cervical spinal stenosis, he was scheduled for a C3-C7 laminoplasty for spondylosis and myelopathy.

Our patient's past medical history was significant for non-insulin dependent diabetes mellitus, asthma, hypertension, benign prostatic hypertrophy, gastro-esophageal reflux disease, deep venous thrombosis, post-traumatic stress disorder, insomnia, chronic pain, and obstructive sleep apnea. For these disorders his medication regimen included metformin, budesonide and formoterol fumarate dihydrate, albuterol, lisinopril, hydrochlorothiazide, tolterodine tartrate, omeprazole, warfarin sodium, gabapentin, zolpidem tartrate, ferrous sulfate, and senna, on a daily basis. He used 900 mg gabapentin three times a day. Our patient did not drink or smoke, and reported allergies to aspirin and peanuts, which caused hives.

On clinical exam, he was alert and oriented in all hemispheres. Cranial nerves II-XII were grossly intact. His peripheral neurological exam revealed decreased sensation to light touch and pinprick in both hands and feet. His muscular strength was 4+/5 in his bilateral deltoids, biceps and triceps, 4/5 in his bilateral wrist extensors, 4/5 in his right finger abductors, 3/5 in his left finger abductors, 4/5 in his bilateral finger flexors, and 4+/5 in his bilateral lower extremities. He showed no pre-operative co-ordination and/or cerebellum abnormalities. Physical examination revealed 3/4 hyper-reflexia in all four extremities. He was Hoffman positive on his right side and Hoffman negative on his left.

Our patient was evaluated by the anesthesia service and medicine services prior to surgery. He did not take metformin, warfarin sodium, or zolpidem tartrate the day before. He did, however, take 900 mg gabapentin up until the morning of his surgery.

Pre-operatively, he was given 1 mg midazolam. He was taken to our operating room and placed under general anesthesia with oral endo-tracheal intubation for C3-C7 laminotomy. Our patient's general anesthetic consisted of an initial 100 mg intubating dose of succinylcholine followed by total intravenous anesthesia (TIVA) using propofol, ketamine, and fentanyl. A total of 100 mg ketamine was used throughout the entire procedure. Neuromuscular monitoring was performed during the case and remained without any significant concerns. Our patient's intra-operative fluid balance included 2400 mL Ringer's Lactate and urine output of 300 mL, with 150 mL blood loss during the three hour and fifty minute case.

Our patient's hemodynamics remained stable throughout the entire case with mean arterial pressures (MAP) maintained at approximately 100 mmHg according to the surgeon's request. At the end of the case, although breathing spontaneously, our patient was not responsive to verbal or noxious stimuli. After being given three separate doses of 40 μg IV naloxone, our patient was moving his arms with anti-gravity strength. He was extubated and taken to the post-anesthesia care unit (PACU) for recovery.

**Figure 2:**
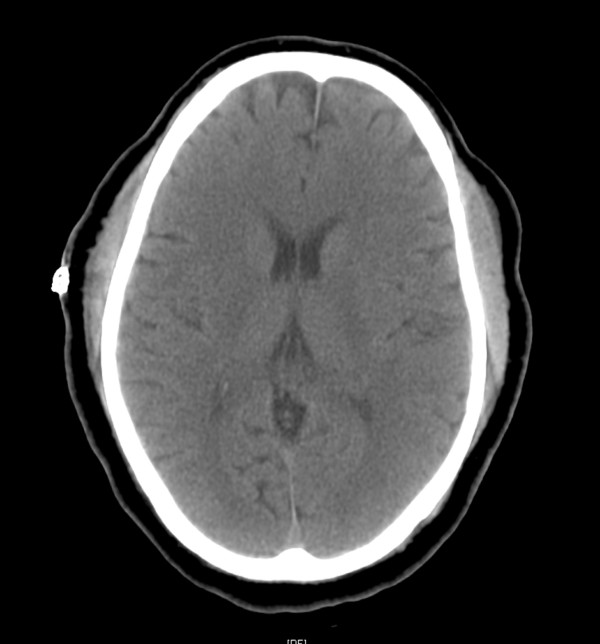
**Post-operative axial CT of head showing no intracranial pathology**.

**Figure 3:**
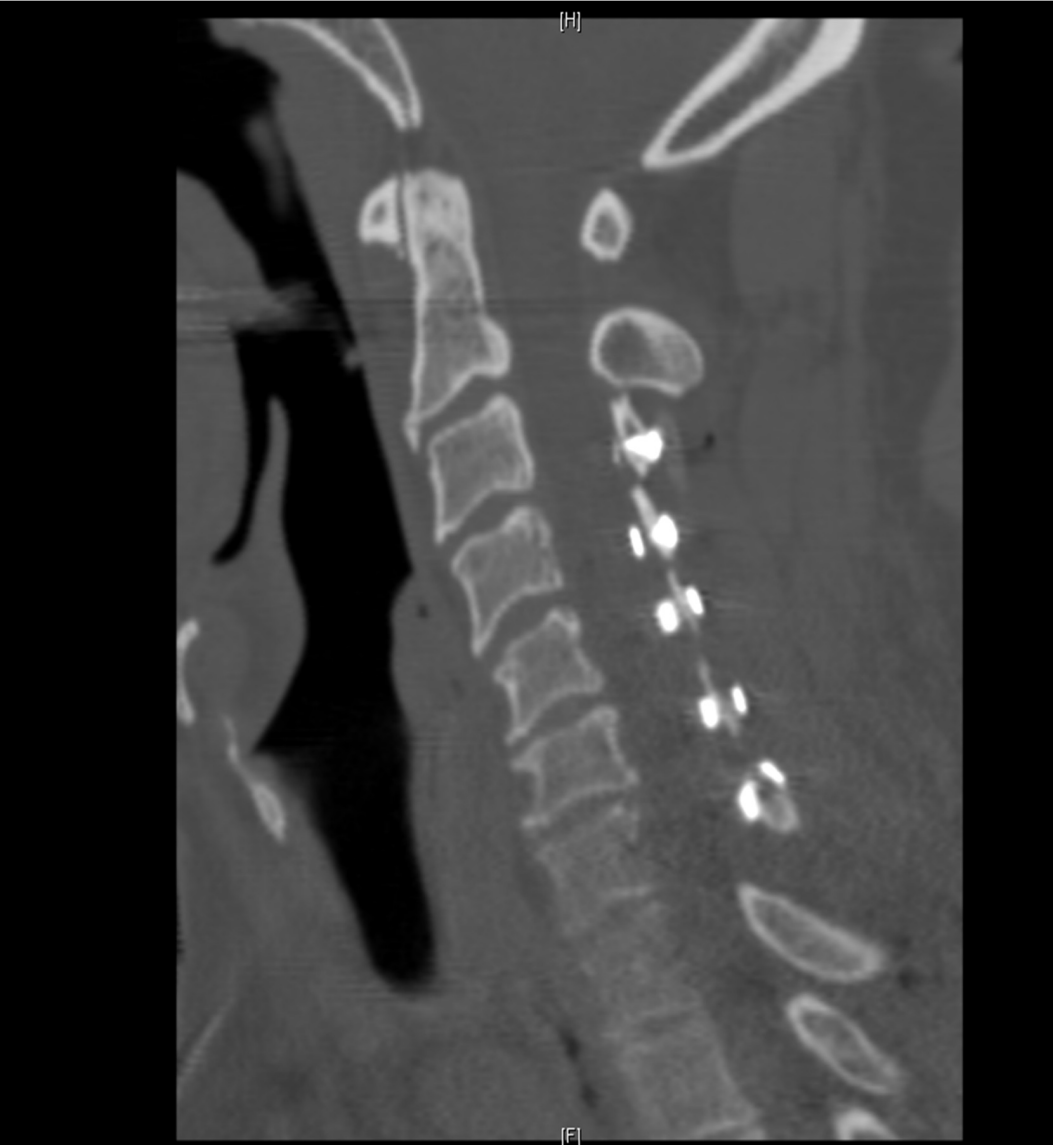
**CT (sagittal view) of cervical region showing normal post-operative changes**.

In the PACU, our patient was not moving his upper or lower extremities and his verbal response consisted of "aha" or blowing through pursed lips. His pupils remained equal and reactive to light. When asked to respond by blinking, our patient would blink uncontrollably. His extremities were flaccid and areflexic. Our patient's vitals remained stable and oxygen saturation was 100%. His arterial blood gas was obtained and was normal, except for partial carbon dioxide (pCO_2_) of 50 mmHg. No electrolyte abnormalities were detected. A urine analysis was within normal limits, along with glucose, ammonia, and lactate levels. Motor evoke potentials were rechecked and remained unchanged from pre-operative values. It was then decided to obtain a CT of the head and neck region (Figures 2 and 3).

**Figure 4:**
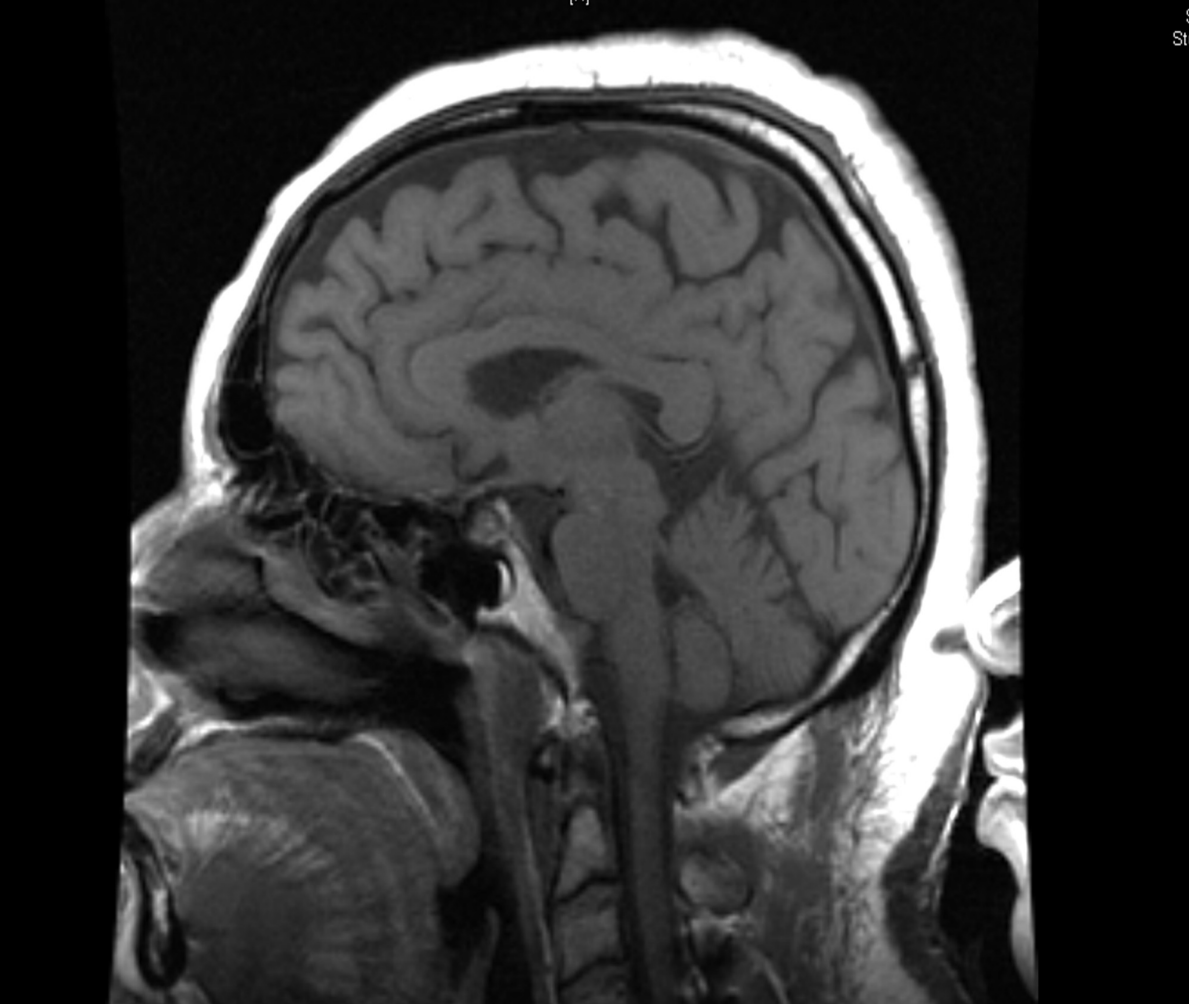
**Normal post-operative sagittal T1-weighted MRI of patient's head**.

**Figure 5:**
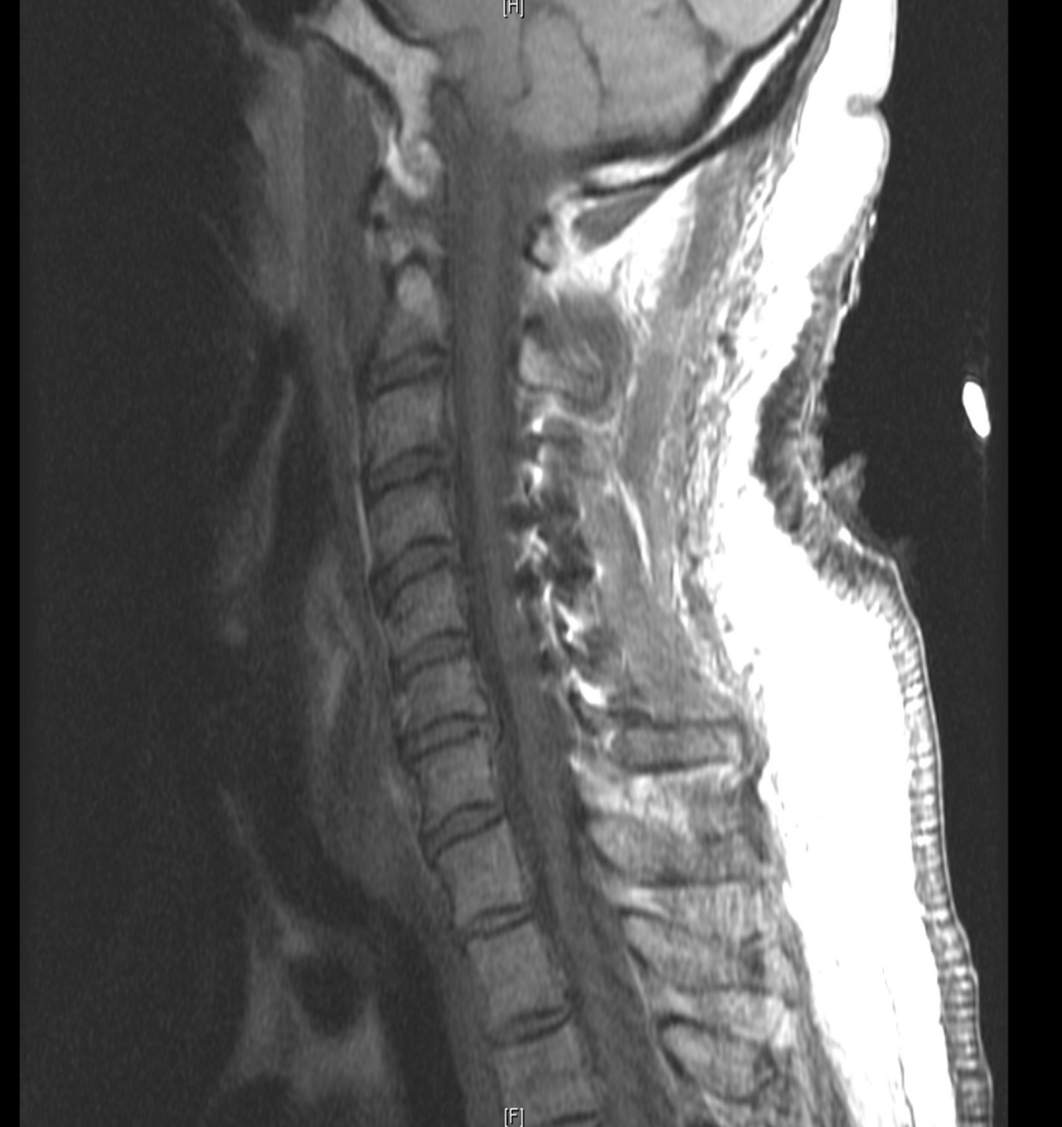
**Normal post-operative sagittal T1-weighted MRI of patient's neck**.

After over two hours of being inappropriately non-responsive to verbal and tactile stimuli in the PACU, our patient was given an additional 400 μg of IV naloxone over a 10 minute period. He was then transferred to the Intensive Care Unit (ICU) for close monitoring and care. MRI and angiography revealed a normal Circle of Willis and no evidence of post-surgical complications in the cervical spine after the C3-C7 decompression (Figures 4 and 5).

In the ICU, blood and imaging studies continued to be unremarkable. Our patient continued to exhibit a dissociative state. During the next four days, however, he showed gradual improvement. He would alternate from being awake and oriented to being dissociative and having decreased sensory and motor function, despite the lack of a toxic or metabolic process. By the fourth day of hospitalization, he had a significant increase in sensory and motor function and was able to answer questions appropriately. The dissociative states became less frequent and of shorter duration. On the ninth day of hospitalization, our patient was discharged home with full recovery and without any objective neurological abnormalities.

## Discussion

Cervical laminectomy and laminoplasty are routine neurosurgical procedures to treat myelopathy secondary to cervical stenosis. According to some studies, cervical laminoplasty has over an 86% success rate [7]. However, even this high success rate is sometimes challenged by other unexplainable factors, such as seen in this case in the post-operative phase [8].

Our patient presented post-operatively in a dissociative state with paralysis, anarthria and preservation of consciousness. He was able to open his eyes to verbal stimuli and preserved eye movement. Several factors were considered, such as prolonged anesthetic effects, inadequate reversal of narcotics, prolonged CNS depression from gabapentin/ketamine interaction, metabolic abnormalities, hypoxia, infection, trauma, hemorrhage, thrombosis leading to infarct, hematoma/abscess in the spinal cord, and/or psychological causes. Organic causes, with the exception of prolonged CNS depression from a gabapentin/ketamine drug interaction, were excluded. However, given our patient's psychiatric history of PTSD, conversion disorder could not be excluded [8].

David Han *et al*. (2007) describe a case in which, after implantation of a spinal cord stimulator, a 42-year-old woman presented with quadriplegia and lower facial diplegia. She was able to open and blink her eyes. There were no organic causes found to explain her condition. A scopolamine patch had been placed prior to surgery. In the PACU the patch was removed and 2 mg physostigmine was given, which produced retching after three minutes and no improvement in neurological symptoms. After ruling out all organic factors, the patient was found to have locked-in syndrome resulting from a conversion disorder. She recovered in less than 24 hours [9].

Locked-in syndrome is defined as quadriplegia and anarthria with the preservation of consciousness. Locked-in syndrome can be categorized into Classic, Incomplete, and Total. Classic is characterized by quadriplegia, anarthria, vertical eye movement, and preserved conscious. Incomplete is the same as classic, except with voluntary movements in addition to vertical eye movement. Total is characterized by a complete loss of mobility and loss of any form of communication, but preservation of consciousness [8-10].

Although conversion disorder remained a plausible answer, prolonged CNS depression from a gabapentin/ketamine drug interaction could not be excluded. This was especially true since our patient had undergone several other surgeries in the past without such a dissociative post-operative recovery. In those cases, however, anesthetics included propofol, fentanyl, and inhalational anesthetic agents. To reiterate, in those previous surgeries, ketamine was not used and our patient did not have a complicated post-operative phase.

As an out-patient, our patient took a high dose of gabapentin (900 mg three times a day) for chronic pain. Gabapentin is structurally related to gamma-aminobutyric acid (GABA), a neurotransmitter that plays an important role in neuronal excitability. The exact mechanism by which gabapentin exerts its properties is not known, however the drug is thought to enhance the release or actions of GABA [11]. According to one study, peri-operative gabapentin can effectively reduce post-operative pain, opioid consumption, and opioid-related adverse effects after surgery. According to the same study, one of the adverse effects of using peri-operative gabapentin is prolonged sedation [12].

Peri-operative ketamine has also been shown to effectively reduce post-operative analgesic requirements. Ketamine is a commonly used anesthetic agent that works as an N-methyl-D-aspartic acid (NMDA) antagonist. NMDA is a synthetic compound that mimics the neurotransmitter glutamate--an excitatory neurotransmitter of the nervous system. Release of glutamate activates post-synaptic glutamate and NMDA receptors. Ketamine is frequently referred to as a dissociative anesthetic, because it interrupts association pathways in the brain. One of the adverse effects of using peri-operative ketamine is prolonged recovery time [13,14].

Together, gabapentin and ketamine could have contributed to the prolonged recovery. An online source relates that these drugs used together may have a synergistic effect and that the patient should be monitored for prolonged CNS and respiratory depression [6]. Several studies have shown how the use of ketamine with other CNS depressants can potentiate CNS depression [15,16].

Our patient took 900 mg gabapentin three times daily up until the morning of his procedure. A total of 100 mg of ketamine was administered during his procedure. Ketamine was selected because of our patient's history of asthma and to reduce his post-operative pain medication requirement. To examine the interaction of these drugs, one must further investigate the bioavailability of these drugs.

Gabapentin elimination half-life is five to seven hours, while the ketamine metabolite half-life is two and a half hours. Gabapentin is eliminated from the systemic circulation by renal excretion. Ketamine is metabolized in the liver and excreted by the kidneys [17,18].

After parenteral administration of ketamine, high concentrations are found in body fat, liver, lung and brain. Peripheral fat distribution, along with redistribution from the CNS to slower equilibrating peripheral tissues, suggests that ketamine is bioavailable beyond its initial peak effect. Nevertheless, ketamine has a wide margin of safety. Unintentional administrations of overdoses of ketamine have been followed by prolonged but complete recovery [18].

## Conclusion

Although this case by itself is not enough evidence to substantiate a true adverse reaction between gabapentin and ketamine, it is enough to warrant further investigation. With the rising numbers of PTSD and chronic pain patients, it is crucial for the clinician to remember cases such as this one if faced with an unusual post-operative recovery phase.

## Consent

Written informed consent was obtained from the patient for publication of this case report and any accompanying images. A copy of the written consent is available for review by the Editor-in-Chief of this Journal.

## Competing interests

The authors declare that they have no competing interests.

## Authors' contributions

AE, RL, and RB were directly involved in the management of the patient's hospital care. All authors listed were major contributors in writing the manuscript. All authors read and approved the final manuscript.
